# A Review of the Effect of Peripheral Amyloid β on the Central Nervous System

**DOI:** 10.3390/cimb48050438

**Published:** 2026-04-23

**Authors:** Zulaikha Elia Zamzuri, Mohd Amir Kamaruzzaman, Seong Lin Teoh, Mohamad Fairuz Yahaya

**Affiliations:** Department of Anatomy, Faculty of Medicine, Universiti Kebangsaan Malaysia, Kuala Lumpur 56000, Malaysia; p147103@siswa.ukm.edu.my (Z.E.Z.); mohdamir@ukm.edu.my (M.A.K.); teohseonglin@ukm.edu.my (S.L.T.)

**Keywords:** Alzheimer’s disease, platelets, blood–brain barrier, amyloid clearance, neuroinflammation

## Abstract

Alzheimer’s disease (AD) is a progressive neurodegenerative disorder classically defined by cerebral amyloid β (Aβ) plaque deposition and tau pathology. In recent years, AD has increasingly been recognized as a multisystem disorder rather than a purely brain-restricted condition, as mounting evidence indicates that Aβ metabolism is a dynamic, bidirectional process involving both central and peripheral compartments. Peripheral tissues, particularly platelets, liver, kidneys, and the gastrointestinal tract, contribute substantially to circulating Aβ levels and influence cerebral amyloid burden. Platelets are now considered the predominant source of peripheral Aβ, accounting for the majority of plasma Aβ under physiological and pathological conditions, while the liver and kidneys play critical roles in Aβ clearance through receptor-mediated uptake, enzymatic degradation and excretion. Disruption of these peripheral clearance pathways elevates circulating Aβ, increasing its transport into the brain via blood–brain barrier (BBB) mechanisms by enhanced RAGE-mediated influx and impaired LRP1-dependent efflux in AD. Peripheral Aβ entry into the central nervous system exacerbates neuroinflammation, mitochondrial dysfunction, and oxidative stress, thereby accelerating neuronal damage and disease progression. This review synthesizes updated evidence on peripheral sources of Aβ, differences between central and peripheral Aβ pools, mechanisms of Aβ transport across the BBB, pathological consequences of peripheral Aβ on the brain and emerging therapeutic strategies targeting peripheral Aβ metabolism, highlighting the importance of a systemic perspective in AD pathogenesis and treatment.

## 1. Introduction

Alzheimer’s disease (AD) is a slow progressive neurodegenerative disorder and the leading cause of dementia worldwide, clinically characterized by memory impairment, cognitive decline, and behavioral disturbances. With global ageing populations, the prevalence of AD is projected to rise dramatically, posing substantial socioeconomic and healthcare challenges, particularly in low- and middle-income countries. Neuropathologically, AD is defined by two major hallmarks: extracellular senile plaques composed predominantly of amyloid β (Aβ) peptides and intracellular neurofibrillary tangles formed by hyperphosphorylated tau protein [1,2,3,4].

Aβ peptides are generated through sequential proteolytic cleavage of the amyloid precursor protein (APP), a type I transmembrane protein widely expressed in neurons as well as in various peripheral tissues. In the amyloidogenic pathway, APP is first cleaved by β-site APP-cleaving enzyme-1 (BACE1), followed by γ-secretase, producing Aβ peptides ranging from 39 to 43 amino acids in length [5,6,7]. Physiological processing of APP also generates neuroprotective fragments via the non-amyloidogenic α-secretase pathway; dysregulation of this balance favors pathological Aβ accumulation in AD. Among these species, Aβ_1–42_ is the most aggregation-prone and neurotoxic, readily forming oligomers and fibrils that impair synaptic plasticity, induce oxidative stress, disrupt mitochondrial function, and promote neuronal death [4,8,9].

For decades, AD was regarded primarily as a brain-restricted disorder driven by neuronal Aβ overproduction. However, over the last decade, a paradigm shift has occurred, with increasing evidence supporting a systemic view of Aβ metabolism, whereby peripheral sources, clearance mechanisms, and transport pathways significantly influence cerebral amyloid burden [3,10,11]. APP and Aβ are expressed not only in the central nervous system (CNS) but also in peripheral tissues such as platelets, liver, kidney, gastrointestinal tract, skin, skeletal muscle, and vascular endothelium [6,12]. This widespread expression suggests that peripheral Aβ pools may act as both reservoirs and modulators of central amyloid pathology.

Importantly, peripheral Aβ is biologically active and capable of interacting dynamically with the brain. Circulating Aβ can cross the blood–brain barrier (BBB) via receptor-mediated transport mechanisms, particularly through the receptor for advanced glycation end-products (RAGE), while efflux of brain-derived Aβ back into the circulation is mediated by transporters such as low-density lipoprotein receptor-related protein-1 (LRP1), ABCA1, and ABCB1 [4,13,14]. In AD, BBB integrity is compromised, RAGE expression is upregulated, and LRP1-mediated clearance is reduced, resulting in a net influx and accumulation of Aβ in the brain [15,16].

In parallel, the steady-state concentration of Aβ in the brain is determined by the balance between production and clearance. Peripheral organs, particularly the liver and kidneys, play essential roles in systemic Aβ clearance, and dysfunction of these organs has been associated with elevated circulating Aβ levels and increased AD risk [10,17,18]. Thus, AD should increasingly be viewed as a disorder involving dysregulated communication between central and peripheral Aβ pools rather than an exclusively CNS-confined pathology.

This review aims to clarify how peripheral Aβ contributes to CNS amyloidosis in AD. Specifically, it focuses on (i) sources of peripheral Aβ, (ii) key differences between central and peripheral Aβ pools, (iii) mechanisms of Aβ transport from the periphery to the brain, (iv) pathological consequences of peripheral Aβ entry into the CNS, and (v) emerging therapeutic strategies targeting peripheral Aβ metabolism. Understanding these interactions may reveal novel diagnostic and therapeutic opportunities for AD.

## 2. Sources of Peripheral Amyloid β

### 2.1. Platelet-Derived Amyloid β

Aβ peptides are generated from APP through enzymatic processing that occurs in a variety of cell types. While neurons are the primary source of Aβ in the brain, platelets represent the dominant source of Aβ in the peripheral circulation. Platelets are anucleate cell fragments derived from megakaryocytes and are highly enriched in APP, accounting for approximately 90% of circulating Aβ in human plasma [12,16]. This disproportionate contribution highlights platelets as a critical interface between vascular biology and amyloid pathology.

Platelets express APP on their plasma membrane and store APP-processing enzymes within α-granules, enabling amyloidogenic processing upon activation [19,20]. Aβ_1–40_ is the predominant species released by platelets, consistent with the dominance of this isoform in peripheral blood. Platelet activation that is triggered by vascular injury, inflammation or oxidative stress can enhance APP cleavage and Aβ secretion, contributing to elevated peripheral Aβ levels [16]. Conditions associated with heightened platelet activation, such as atherosclerosis, diabetes mellitus and chronic inflammation, are therefore likely to amplify peripheral Aβ production.

Pathological alterations in platelet APP processing have been consistently reported in AD patients, including altered APP isoform ratios and increased secretase activity favoring Aβ production [19,20]. Beyond Aβ generation, platelets actively promote Aβ aggregation as in vitro studies demonstrate that platelets can convert soluble Aβ_1–40_ into higher-order oligomers and fibrillar aggregates with increased neurotoxicity. These platelet-associated aggregates may exhibit enhanced resistance to enzymatic degradation, prolonging their circulation time and increasing their pathogenic potential [12,21].

At the molecular level, platelet surface receptors such as integrin αIIbβ3 and glycoprotein VI (GPVI) have been identified as binding partners for Aβ_1–40_, facilitating platelet activation and aggregation [20]. This interaction establishes a feed-forward loop in which platelet activation promotes further Aβ release and aggregation. Importantly, activated platelets also secrete pro-inflammatory mediators and reactive oxygen species (ROS) that impair endothelial function and BBB integrity, thereby facilitating transport of peripheral Aβ into the brain [15,16].

Experimental studies demonstrate a positive correlation between peripheral Aβ levels and cerebral amyloid burden, supporting the concept that platelet-derived Aβ contributes to AD pathogenesis [19]. Collectively, these findings identify platelets as a critical mechanistic link between peripheral Aβ metabolism, vascular dysfunction, and central amyloid pathology.

### 2.2. Peripheral Tissues and Organs

In addition to platelets, multiple peripheral tissues and organs express APP and contribute to systemic Aβ production and clearance. APP expression has been detected in the liver, kidneys, adrenal glands, heart, pancreas, skeletal muscle, spleen, gastrointestinal tract, skin, and vascular endothelial cells [3,10].

The liver plays a central role in peripheral Aβ metabolism. Hepatocytes express high levels of LRP1, enabling efficient receptor-mediated uptake and degradation of circulating Aβ [10,18]. Experimental studies have shown that hepatogenic Aβ associates with triglyceride-rich lipoproteins, enters the circulation, and can subsequently deposit in the brain, inducing AD-like neuropathology including neurodegeneration and brain atrophy [6]. Clinical and epidemiological evidence further links liver dysfunction, including non-alcoholic fatty liver disease (NAFLD), with elevated plasma Aβ levels and increased cerebral amyloid accumulation [17].

The gastrointestinal tract has emerged as a potential contributor to peripheral Aβ dynamics. APP expression and Aβ immunoreactivity have been detected in intestinal epithelial cells and enteric neurons in both AD patients and experimental models, supporting the presence of amyloid-related pathology outside the CNS [6,22]. These findings suggest that the gut may serve as a peripheral source of Aβ under certain pathological conditions. Intestinal inflammation and barrier dysfunction may facilitate the entry of locally produced Aβ into the systemic circulation through disruption of the gut-vascular barrier, thereby increasing circulating amyloid burden [22]. In addition, interactions between Aβ, gut microbiota composition and mucosal immune signaling may further influence systemic amyloid levels and neuroinflammatory responses via the gut–brain axis [22]. While the quantitative contribution of intestinal Aβ to circulating pools remains to be fully defined, accumulating evidence supports a potential gut-derived component of peripheral amyloid burden that may modulate central pathology [6,10,22]. Beyond liver and gut, Aβ deposition has been reported in skin, skeletal muscle, cardiac tissue, and ocular structures such as the retina and lens [12]. In many cases, these deposits are believed to originate from circulating Aβ rather than local overproduction, reinforcing the concept of AD as a systemic amyloid disorder rather than a purely CNS-limited disease.

### 2.3. Differences Between Central and Peripheral Amyloid β Pools

Despite a shared origin from APP, substantial differences exist between central and peripheral Aβ pools in terms of molecular composition, concentration, and biological behavior. Recent quantitative proteomic and biochemical analyses demonstrate that these differences reflect distinct biological states of Aβ depending on tissue context and clearance dynamics [3,23]. One key distinction lies in the relative abundance of Aβ species. In the brain, Aβ_1–42_ predominates and constitutes the core component of senile plaques due to its high aggregation propensity and neurotoxicity. In contrast, the peripheral circulation is dominated by Aβ_1–40_, which is more soluble and less prone to aggregation [3,23]. The molecular weight of Aβ_1–40_ is 4330 g/mol, whereas the longer Aβ_1–42_ peptide has a computed molecular weight of 4514.0 g/mol [24,25].

**Figure 1:**
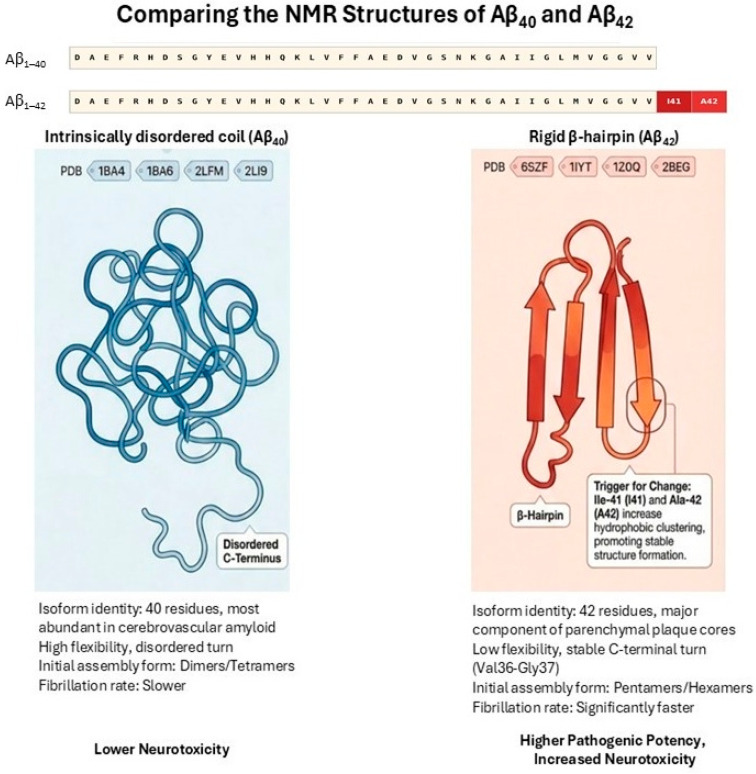
Structural comparison of Aβ_40_ and Aβ_42_ monomers in solution. In aqueous solution, Aβ_40_ exists primarily as a flexible and disordered “collapsed coil” without a stable C-terminal structure. Representative NMR structures include PDB IDs 1BA4, 1BA6, and 1HZ3. In contrast, the two extra C-terminal residues in Aβ_42_ (Ile41 and Ala42) cause extensive hydrophobic clustering, forcing the peptide into a more rigid and structured β-hairpin fold. Representative NMR structures for Aβ_42_ include PDB IDs 1IYT and 6SZF. The stable β-hairpin geometry of Aβ_42_ restricts its overall flexibility and acts as a direct building block for the rapid assembly of toxic oligomers and mature amyloid fibrils, underlying its significantly higher neurotoxicity compared to Aβ_40_. (Adapted from [24,25,26,27,28] and partly created with assistance from an AI-based visualization tool (NotebookLM (Apr 2026 version) [Large language model]. https://notebooklm.google.com/)).

Aβ_1–42_ differs from Aβ_1–40_ by the presence of two additional C-terminal residues, isoleucine-41 and alanine-42, which substantially increase hydrophobicity and β-sheet stabilization. Recent analyses of Nuclear Magnetic Resonance (NMR) solution structures deposited in the Protein Data Bank (RCSB-PDB) highlight distinct conformational trends between the two peptides that dictate these aggregation properties [21,26]. In aqueous solution, the Aβ_1–40_ monomer lacks a stable C-terminal secondary structure and predominantly behaves as a highly flexible, intrinsically disordered “collapsed coil” (representative PDB IDs: 1BA4, 1BA6, 1HZ3) [21,27]. In contrast, the additional hydrophobic residues in Aβ_1–42_ drive extensive intramolecular hydrophobic clustering, causing the C-terminus to adopt a more rigid and structured β-hairpin fold—often pairing residues 31–34 and 38–41 (representative PDB IDs: 1IYT, 6SZF) [21,26,28,29]. This C-terminal rigidity actively restricts the conformational space Aβ_1–42_ can explore, acting as an ideal geometric building block for rapid assembly into toxic oligomers (paranuclei) and mature amyloid fibrils [26,27] (Figure 1). Structural studies demonstrate that these residues promote intermolecular interactions within the C-terminal region, enhancing oligomerization propensity and accelerating fibrillogenesis [30,31]. Cryo-electron microscopy analyses further reveal distinct fibril architectures between Aβ_1–42_ and Aβ_1–40_, with the latter forming more compact and neurotoxic conformations [32]. These structural differences underlie the higher aggregation kinetics, increased oligomer stability and greater neurotoxicity observed for Aβ_1–42_ compared to Aβ_1–40_. Although Aβ_1–42_ is the predominant species within parenchymal plaques due to its higher aggregation propensity and fibrillogenic potential [21], Aβ_1–40_ constitutes the majority of circulating Aβ and remains biologically active [12,18,33]. The lower intrinsic aggregation tendency of Aβ_1–40_ does not preclude its pathogenic relevance. Circulating Aβ_1–40_ can cross the BBB via RAGE-mediated transport mechanisms [13,14], and preferentially accumulates in cerebral vasculature, where it is strongly associated with cerebral amyloid angiopathy and endothelial dysfunction [16,21]. Moreover, experimental studies demonstrate that Aβ_1–40_ can co-aggregate with Aβ_1–42_ and modulate nucleation kinetics, thereby influencing fibril formation and vascular amyloid deposition [21]. Importantly, elevated peripheral Aβ_1–40_ increases the total systemic amyloid burden and may shift the influx–efflux equilibrium at the BBB toward net cerebral accumulation, particularly in the context of impaired LRP1-mediated clearance [13,18,34]. Thus, peripheral Aβ_1–40_ contributes to CNS pathology not primarily as a plaque-dominant species, but as a vascular, transport-modulating, and aggregation-promoting factor.

Several mechanisms may account for this divergence. Differential expression of APP isoforms and tissue-specific γ-secretase activity between CNS and peripheral tissues may lead to distinct Aβ cleavage patterns [3]. In addition, the peripheral microenvironment contains abundant Aβ-binding proteins, including albumin and lipoproteins, as well as Aβ-binding cells such as erythrocytes, which facilitate Aβ sequestration, transport, and clearance [10,18].

Central Aβ concentrations are also substantially higher than peripheral levels. This reflects both increased neuronal production and relatively inefficient clearance within the brain, exacerbated by age-related BBB dysfunction. In contrast, peripheral Aβ is diluted within the large volume of the circulatory system and efficiently cleared by the liver, kidneys, and peripheral immune cells [17,18]. These factors help explain why extensive Aβ aggregation occurs primarily in the brain and cerebral vasculature, with comparatively limited deposition in peripheral organs.

### 2.4. Tools for Investigating Peripheral Amyloid β

Peripheral Aβ is increasingly being recognized as a biomarker, a compartment accessible to systemic clearance, transport and peripheral-central crosstalk in AD. Quantitative tests like Enzyme Immunoassay (ELS), Electrochemiluminescence and single molecule array (SIMOA) can be used for strong detection of plasma Aβ_40_ and Aβ_42_, while immunoprecipitation mass spectrometry (IP-MS) can provide better analytical specificity and precision of Aβ_42_/Aβ_40_ ratio [33,35,36]. Structural and aggregation analyses, such as Thioflavin-T binding, circular dichroism, dynamic light scattering, atomic force microscopy and transmission electron microscopy (TEM), are used for the characterization of oligomeric and beta-sheet conformations [37,38,39,40]. Cellular localization and interaction with immune cells can be determined through the use of immunohistochemistry, confocal imaging and flow cytometry of platelets and PBMCs [41]. Functional assays are used to interrogate BBB transport and receptor-mediated clearance pathways through LRP1, LDLR and RAGE [34]. Extracellular vesicles provide an additional peripheral carrier that allows for the characterization of neuron-derived Aβ cargo [42]. Clinically, there is support for translational utility and early detection from plasma Aβ_42_/Aβ_40_ ratios and plasma-PET concordance [43]. Collectively, these tools offer complementary quantitative, structural and mechanistic insight into the peripheral Aβ dynamics relevant to biomarker development for AD (Table 1).

## 3. Mechanisms of Amyloid β Transport from Periphery to Brain

The BBB is a highly specialized and selectively permeable interface that regulates molecular exchange between the systemic circulation and CNS. It is composed primarily of brain microvascular endothelial cells interconnected by tight junctions, supported by pericytes, astrocytic end-feet, and the extracellular matrix [44,45]. Under physiological conditions, the BBB tightly restricts entry of macromolecules such as Aβ; however, in AD, BBB integrity and transport functions are profoundly altered [13,14].

The BBB regulates amyloid β (Aβ) trafficking through a coordinated network of transporters that balance influx and efflux across the cerebral endothelium [45,46]. Circulating Aβ can enter the brain via the receptor for advanced glycation end products (RAGE), which is upregulated in aging and Alzheimer’s disease and facilitates receptor-mediated influx of peripheral Aβ into the CNS [13,47]. In contrast, LRP1 mediates Aβ efflux from the abluminal endothelial surface into the circulation and plays a central role in maintaining cerebral Aβ homeostasis; ageing-associated oxidative stress, metabolic dysregulation, and endothelial dysfunction reduce LRP1 expression and transport efficiency, thereby promoting cerebral Aβ accumulation [48,49]. Recent therapeutic strategies that enhance LRP1-mediated transport demonstrate the functional importance of this pathway in clearing Aβ and restoring cognitive function [50]. In addition, apolipoprotein E (ApoE) has been shown to modulate blood–brain barrier permeability and influence Aβ transport dynamics, with important implications for Aβ_1–42_ trafficking across the BBB [51]. The luminal ATP-binding cassette transporter ABCB1 (P-glycoprotein) also contributes to Aβ export across the BBB and reduced ABCB1 expression or activity, which has been observed in ageing and Alzheimer’s disease, is associated with increased amyloid deposition [52,53,54]. Additionally, ABCA1 can enhance ApoE lipidation and thereby promote Aβ clearance and modulate vascular and inflammatory pathways relevant to AD pathology [55].

Beyond transendothelial transport, perivascular clearance pathways which include intramural periarterial drainage and the glymphatic system contribute substantially to CNS Aβ elimination. Perivascular drainage and glymphatic systems play key roles in interstitial solute clearance, including Aβ, and dysfunction of these pathways is implicated in AD pathology [56]. Collectively, dysfunction of these integrated transport and clearance systems shifts the influx–efflux equilibrium toward cerebral Aβ retention and progressive amyloid pathology.

Conversely, efflux of brain-derived Aβ into the peripheral circulation is mediated primarily by LRP1, along with ATP-binding cassette transporters such as ABCA1 and ABCB1 (P-glycoprotein). LRP1 is abundantly expressed on the abluminal surface of BBB endothelial cells and plays a critical role in clearing soluble Aβ from the brain interstitial fluid into the blood [13,18]. In AD, LRP1 expression and function are reduced, resulting in impaired Aβ clearance and a net shift toward accumulation within the CNS [34].

BBB dysfunction further exacerbates peripheral to central Aβ transport. Experimental studies demonstrate that Aβ itself disrupts BBB integrity by altering tight junction proteins, including zonula occludens-1 (ZO-1), claudin-5 and occludin. In an in vitro BBB model using murine cerebral endothelial cells, exposure to Aβ_1–42_ particularly in its oligomeric form induced dose-dependent increases in BBB permeability, upregulated RAGE expression, and downregulated tight junction scaffolding proteins [14]. These effects were more pronounced with Aβ oligomers than with monomeric or fibrillar forms, underscoring the pathogenic potency of soluble Aβ species [21].

Peripheral inflammatory processes also play a critical role in modulating BBB permeability. Activated platelets and innate immune cells release cytokines, chemokines, ROS and proteolytic enzymes that promote endothelial senescence and junctional disassembly [15,16]. Systemic inflammation amplifies BBB permeability by inducing endothelial oxidative stress and downregulating tight junction proteins, thereby facilitating peripheral Aβ entry into the CNS even in preclinical stages of AD [57,58]. Platelet-derived Aβ and inflammatory mediators can synergistically impair BBB function, thereby facilitating increased translocation of circulating Aβ into the brain.

**Figure 2:**
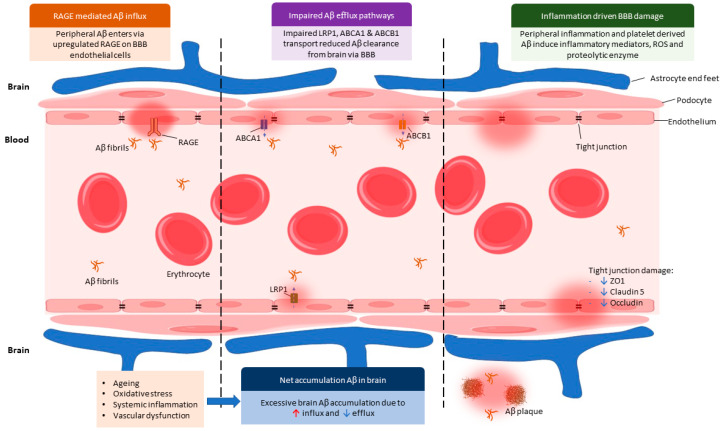
Cellular Mechanisms of Peripheral Aβ Trafficking and BBB Dysfunction in AD: Peripheral Aβ enters the brain via upregulated RAGE on BBB endothelial cells, promoting increased influx. Impairment of efflux transporters, including LRP1, ABCA1, and ABCB1, reduces Aβ clearance from the brain. Concurrently, peripheral inflammation and platelet-derived Aβ stimulate inflammatory mediators, reactive oxygen species, and proteolytic enzymes, leading to endothelial activation and barrier disruption. Tight junction proteins (ZO-1, claudin-5, and occludin) are reduced, increasing BBB permeability. Ageing, oxidative stress, systemic inflammation, and vascular dysfunction further exacerbate these alterations. The combined increase in influx and decrease in efflux results in net Aβ accumulation in the brain, facilitating plaque formation and contributing to progressive neuroinflammatory and neurodegenerative processes characteristic of AD. Some images were taken from Biosketch.art, available at: https://biosketch.art (accessed on: 8 February 2026).

Collectively, these findings support a model in which peripheral Aβ gains access to the CNS through a combination of enhanced RAGE-mediated influx, impaired LRP1-dependent efflux, and BBB structural disruption. This imbalance is further amplified by ageing, systemic inflammation, and vascular dysfunction, all of which are prominent risk factors for AD (Figure 2).

## 4. Effects of Amyloid β Transport from Periphery to Brain

### 4.1. Neuroinflammation

Neuroinflammation is a central pathological feature of AD and plays a critical role in disease initiation and progression. It is characterized by sustained activation of microglia and astrocytes, leading to the release of pro-inflammatory cytokines, including tumor necrosis factor-α (TNF-α), interleukin-1β (IL-1β), interleukin-6 (IL-6), interferon-γ (IFN-γ), as well as chemokines, complement proteins and ROS [4,59,60]. Accumulating evidence indicates that peripheral Aβ significantly contributes to this inflammatory milieu following its entry into the CNS [15].

Microglia, which are the resident immune cells of the brain, are highly sensitive to Aβ. Under physiological conditions, microglia contribute to Aβ clearance through phagocytosis and degradation. Activation of microglia can be classified into the M1 phenotype which is pro-inflammatory and the M2 phenotype which is immunosuppressive [60,61]. Thus, exposure to elevated levels of Aβ particularly oligomeric forms drives microglial activation toward a pro-inflammatory M1 phenotype. M1-polarized microglia secrete TNF-α, IL-1β, IL-6, and inducible nitric oxide synthase (iNOS), all of which exacerbate neuronal injury and synaptic dysfunction [6,62]. Peripheral Aβ exposure has been shown to prime microglia toward a pro-inflammatory phenotype, lowering their activation threshold and exacerbating neuroinflammatory responses upon subsequent CNS Aβ accumulation [63].

Aβ exhibits structural and functional similarities to antimicrobial peptides, enabling it to activate innate immune signaling pathways. Aggregated Aβ can insert into cell membranes and form pore-like structures, triggering ionic imbalance and danger-associated molecular pattern (DAMP) signaling that further activates glial cells [4,64]. This immune activation is not restricted to brain-derived Aβ as peripheral Aβ entering the CNS is equally capable of initiating these responses.

Importantly, peripheral immune activation precedes and amplifies central neuroinflammation. Shi et al. [15] demonstrated that circulating Aβ activates peripheral innate immune cells, which release inflammatory mediators that increase BBB permeability during early stages of AD. As the disease progresses, peripheral immune cells infiltrate the brain parenchyma and localize around Aβ plaques, where they interact with resident microglia to amplify inflammatory signaling. This creates a self-perpetuating cycle in which peripheral and central immune responses reinforce each other, accelerating neurodegeneration.

Astrocytes also contribute to Aβ-driven neuroinflammation. Reactive astrocytes surrounding Aβ plaques secrete cytokines and reduce metabolic and trophic support to neurons, further compromising neuronal survival [65]. Together, these processes establish chronic neuroinflammation as a key downstream consequence of peripheral Aβ entry into the CNS.

### 4.2. Mitochondrial Dysfunction and Oxidative Stress

Emerging evidence indicates that Aβ localizes preferentially to neuronal mitochondria, where it disrupts mitochondrial dynamics, impairs calcium handling and enhances ROS production, events that precede overt plaque deposition [3,66]. Neurons are particularly vulnerable to mitochondrial impairment due to their high energy demands and reliance on oxidative phosphorylation for ATP production. Increasing evidence suggests that Aβ derived from both central and peripheral sources directly targets mitochondrial structure and function [4,8].

Soluble Aβ oligomers can accumulate within neuronal mitochondria, where they disrupt the balance between mitochondrial fission and fusion, impair electron transport chain activity, and reduce ATP synthesis. Aβ has been shown to interact with mitochondrial proteins such as amyloid-binding alcohol dehydrogenase (ABAD), leading to altered redox homeostasis and enhanced production of ROS [4].

Mitochondria are the principal source of cellular ROS, generated through electron leakage at complexes I and III of the electron transport chain. In AD, damaged mitochondria become less efficient at ATP production while simultaneously generating excessive ROS, accounting for up to 90% of intracellular oxidative stress [8]. Elevated ROS levels induce oxidative damage to lipids, proteins, and nucleic acids, further compromising neuronal integrity.

Oxidative stress is strongly associated with Aβ burden. Elevated levels of Aβ_1–40_ and Aβ_1–42_ correlate with increased markers of protein oxidation, lipid peroxidation, and DNA damage in vulnerable brain regions such as the hippocampus and cortex, whereas brain regions with low Aβ deposition, such as the cerebellum, exhibit comparatively minimal oxidative damage [8]. Peripheral Aβ that enters the brain adds to this oxidative burden, reinforcing mitochondrial dysfunction.

Moreover, oxidative stress may impair Aβ clearance mechanisms. Oxidative modification of LRP1 has been proposed to reduce its Aβ-binding capacity, leading to diminished efflux of Aβ from the brain and further accumulation of neurotoxic species [8,13]. Thus, mitochondrial dysfunction, oxidative stress, and Aβ accumulation form a tightly interconnected pathological triad that drives neuronal degeneration in AD.

## 5. Peripheral Organ Contribution to Central Nervous System Amyloid β Burden

Beyond the brain and BBB, peripheral organs play essential roles in maintaining systemic Aβ homeostasis. Increasing evidence indicates that dysfunction of peripheral clearance mechanisms significantly contributes to elevated circulating Aβ levels, thereby increasing the amount of Aβ available for transport into the CNS and exacerbating AD pathology [10,18].

### 5.1. Liver

The liver is widely regarded as the principal organ responsible for peripheral Aβ clearance. Hepatocytes express high levels of LRP1, which mediates receptor-dependent uptake and degradation of circulating Aβ [17,18]. Once internalized, Aβ can be enzymatically degraded within hepatocytes or excreted into bile, thereby reducing systemic Aβ burden.

Experimental studies have demonstrated that impairment of hepatic Aβ clearance leads to increased plasma Aβ levels and enhanced cerebral amyloid deposition. Conversely, enhancement of hepatic LRP1 expression accelerates peripheral Aβ removal and reduces brain Aβ accumulation [10,23]. Notably, Aβ effluxed from the brain is transported in association with high-density lipoprotein (HDL) particles, highlighting the integration of lipid metabolism with Aβ clearance pathways [18].

Recent human and animal studies support a liver–brain axis in AD, demonstrating that impaired hepatic clearance of circulating Aβ results in increased brain amyloid deposition and cognitive decline [67,68]. Patients with liver dysfunction, including cirrhosis and hepatocellular carcinoma, exhibit elevated plasma Aβ levels [17]. Moreover, non-alcoholic fatty liver disease (NAFLD) has been linked to increased cerebral Aβ deposition and cognitive impairment in both human and animal studies. NAFLD-associated inflammation and lipid dysregulation impair LRP1-mediated hepatic Aβ clearance, thereby promoting systemic and central amyloid accumulation [17].

### 5.2. Kidney

The kidneys also contribute significantly to peripheral Aβ clearance through filtration and excretion. Radiotracer studies using intravenously or intracranially administered radiolabeled Aβ have demonstrated subsequent accumulation of radioactivity in renal tissue and urine, indicating that Aβ is filtered from the blood by the kidneys [18].

Experimental and clinical studies provide further support for renal involvement in Aβ homeostasis. Tian et al. [69] demonstrated that unilateral nephrectomy in animal models resulted in elevated plasma and brain Aβ levels, accompanied by worsened cognitive performance and enhanced AD-like pathology. In humans, Aβ has been detected in renal tissue and urine, and levels of Aβ in renal arterial blood exceed those in renal venous blood, indicating net renal clearance of circulating Aβ.

Renal dysfunction has been associated with increased dementia risk and elevated plasma Aβ concentrations. Even moderate renal impairment may compromise systemic Aβ clearance, thereby indirectly increasing cerebral amyloid burden [18]. Further, chronic kidney disease has been associated with elevated plasma Aβ levels and increased risk of cognitive impairment, suggesting that even subclinical renal dysfunction may contribute to systemic amyloid accumulation [70,71]. These findings suggest that kidney health is an important but often overlooked factor influencing AD pathogenesis.

### 5.3. Gastrointestinal Tract and Other Peripheral Tissues

Beyond liver and kidney clearance, the gastrointestinal system may influence systemic Aβ homeostasis through coordinated gut–liver–brain axis interactions that integrate metabolic, inflammatory, and vascular pathways [6,10,22,67]. Rather than serving solely as a production site, intestinal dysfunction may modulate circulating Aβ levels indirectly via increased epithelial permeability, systemic inflammation and microbiota-mediated immune signaling [22,58]. Disruption of the gut-vascular barrier can enhance the translocation of inflammatory mediators and potentially amyloidogenic peptides into the blood-stream, thereby increasing the circulating amyloid pool available for BBB transport [22]. Emerging evidence further indicates that systemic inflammatory signaling originating from the gut can impair BBB integrity and influence cerebral amyloid accumulation [58,72]. Collectively, these findings suggest that intestinal health may influence cerebral amyloidosis primarily through systemic regulatory and vascular mechanisms rather than direct plaque formation within the gut.

## 6. Therapeutic Implications: Targeting Peripheral Amyloid β

Despite years of research, the development of effective drug therapies for AD has been challenging, mostly due to the multifactorial etiologies of the disorder that can initiate neurodegeneration interdependently. However, current evidence suggests that combination therapy targeting several factors simultaneously appears to be promising [73]. Given the growing recognition of peripheral Aβ as a contributor to CNS pathology, targeting peripheral Aβ metabolism has emerged as a promising therapeutic strategy for AD. Compared with direct CNS-targeted approaches, peripheral interventions may offer improved safety, accessibility, and feasibility.

### 6.1. Peripheral Immunotherapy

**Figure 3:**
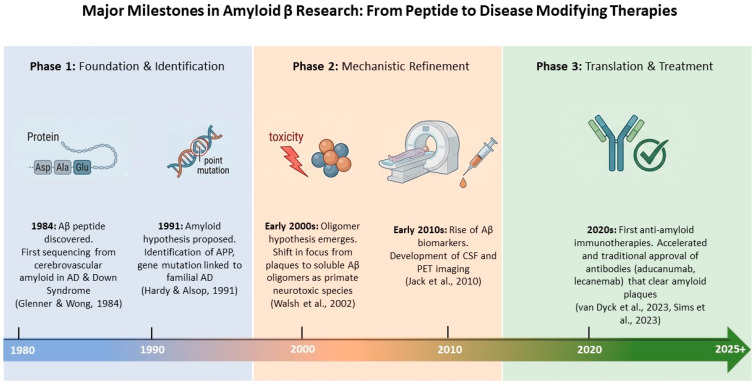
Major Milestones in Aβ Research: From Peptide Identification to Disease-Modifying Therapies. This timeline categorizes the progression of AD research into three distinct phases. Phase 1 (1980s–1990s) focuses on the foundational discovery of the Aβ peptide and the genetic linking of APP mutations to familial AD [74,75]. Phase 2 (2000s–2010s) marks the mechanistic shift toward soluble Aβ oligomers as the primary neurotoxic species and the rise in CSF and PET imaging biomarkers [76,77]. Phase 3 (2020s–2025+) represents the successful translation into clinical practice with the FDA approval of immunotherapies such as lecanemab and donanemab that effectively clear amyloid plaques and slow cognitive decline [78,79] (Some images were created with assistance from an AI-based visualization tool (NotebookLM (Apr 2026 version) [Large language model]. https://notebooklm.google.com/).

Immunotherapy aimed at sequestering Aβ in the periphery has demonstrated efficacy in reducing brain amyloid burden in experimental models. This therapeutic evolution is the culmination of over forty years of research milestones (Figure 3). Since the discovery and sequencing of the Aβ peptide in 1984 [74], the field has transitioned from basic mechanistic refinement, such as the proposal of the Amyloid Hypothesis [75] and the identification of soluble oligomers [76], to the current era of clinical translation. The development of ultrasensitive biomarkers [77] and the recognition of the ‘peripheral sink’ effect were critical for this progress, eventually leading to the 2020s milestone: the clinical approval of anti-amyloid monoclonal antibodies like aducanumab, lecanemab and donanemab [78,79]. These therapies validate the importance of targeting Aβ clearance, whether through direct central action or by modulating peripheral-central Aβ dynamics.

Peripheral administration of these anti-Aβ antibodies facilitates the binding of circulating Aβ, effectively lowering free plasma levels and establishing a concentration gradient that encourages the efflux of Aβ from the brain into the systemic circulation, a mechanism known as the ‘peripheral sink’ effect [11]. This concept is predicated on a dynamic equilibrium existing between central and peripheral amyloid compartments, where systemic reduction promotes a net outward movement of brain-derived Aβ to enhance overall clearance [10,23]. Furthermore, peripheral Aβ pools interact closely with systemic immune and metabolic pathways, suggesting that these extra-cerebral dynamics can modulate internal brain homeostasis and contribute to therapeutic outcomes beyond simple direct CNS targeting [15].

The recent clinical introduction of monoclonal antibodies such as aducanumab, lecanemab, and donanemab marks a turning point in this field. While these agents primarily target aggregated CNS amyloid to enhance microglial-mediated phagocytosis and plaque disaggregation, they also facilitate the transport of Aβ from the cerebrospinal fluid into the plasma [80]. Although these therapies primarily target CNS amyloid, increasing evidence suggests that their effects on peripheral Aβ dynamics contribute to their overall efficacy. Aβ-targeting therapeutics, particularly monoclonal antibodies and strategies enhancing peripheral clearance pathways, may influence peripheral amyloid dynamics in addition to central effects. The peripheral sink hypothesis posits that lowering circulating Aβ can shift the equilibrium across the BBB toward net efflux from the brain, thereby reducing cerebral amyloid burden. Several monoclonal antibodies targeting aggregated Aβ such as aducanumab, donanemab, and lecanemab have demonstrated the capacity to reduce cerebral amyloid burden in clinical trials and slow clinical progression [78,79,81]. Emerging strategies also include enhancing peripheral clearance mechanisms such as upregulation of hepatic LRP1, increasing enzymatic degradation of circulating Aβ (e.g., neprilysin enhancers), or immunotherapies directed at peripheral Aβ species to augment elimination and reduce systemic amyloid load [10,23]. These approaches highlight that therapeutic modulation of peripheral Aβ pools not only serves as an adjunct to central amyloid lowering but may also contribute independently to altering disease progression.

### 6.2. Enhancing Peripheral Clearance via LRP1

A study by Shaikh et al. [82] has shown that exploring alternative approaches, such as natural compounds like Kelulut honey (KH), may be valuable for both preventive and therapeutic interventions for AD. In this context, targeting LRP1-mediated peripheral Aβ clearance represents another promising therapeutic avenue, as natural compounds and several pharmacological agents have been shown to enhance LRP1 expression and activity in preclinical models, although no LRP1-specific therapies have yet received clinical approval.

Plant-derived compounds such as *Withania somnifera* have been reported to upregulate hepatic LRP1 expression and reduce cerebral Aβ levels in AD mouse models. In addition, metabolic drugs such as rosiglitazone increase hepatic LRP1 expression through peroxisome proliferator-activated receptor-γ (PPARγ)-dependent pain pathways, enhancing peripheral Aβ clearance in vivo [23].

Statins have also attracted attention for their potential role in modulating LRP1 expression. Atorvastatin has been shown to regulate hepatic LRP1 via sterol regulatory element-binding protein-2 (SREBP-2), leading to improved Aβ clearance [23]. Consistent with these mechanistic findings, a large systematic review and meta-analysis reported that statin use is associated with a reduced risk of developing dementia and AD [83].

### 6.3. Peripheral Enzymatic Degradation

Enhancing peripheral Aβ degradation through proteolytic enzymes represents an additional therapeutic strategy. Neprilysin (NEP), a major Aβ-degrading enzyme, plays a critical role in Aβ catabolism. Experimental studies demonstrate that sustained expression of NEP in skeletal muscle or increased circulating NEP levels significantly reduces brain Aβ burden and improves cognitive performance in AD models [10,83].

Similarly, peripherally derived angiotensin-converting enzyme (ACE)-enhanced macrophages have been shown to alleviate AD pathology and behavioral deficits by promoting Aβ degradation [10,84]. These findings suggest that strengthening peripheral degradation pathways may effectively complement central Aβ clearance mechanisms.

Importantly, peripheral targeting strategies may offer a safer alternative to direct CNS interventions, reducing the risk of adverse effects such as amyloid-related imaging abnormalities (ARIA) associated with intracerebral amyloid removal.

### 6.4. Diagnostic Reliability

Peripheral Aβ offers practical and translational advantages over central Aβ measures. Peripheral sampling (plasma, serum, EVs) is minimally invasive and scalable, enabling longitudinal monitoring and population-level screening. Peripheral Aβ_42_/Aβ_40_ ratios show early biomarker potential and can support trial enrichment and therapeutic response assessments. Moreover, peripheral Aβ reflects systemic clearance, vascular transport, immune interactions, and blood–brain barrier exchange, thereby capturing pathophysiological dimensions not accessible through CSF or PET alone.

Recent advances in ultrasensitive detection platforms, including immunoprecipitation–mass spectrometry (IP-MS) and single-molecule array (SIMOA) technologies, have significantly improved the clinical utility of plasma Aβ measurements. Studies have reported that plasma Aβ_42_/Aβ_40_ ratios measured by IP-MS achieve area-under-the-curve (AUC) values ranging from 0.85 to 0.94 for predicting amyloid PET positivity [33,85]. Lower limits of detection in the low picogram per milliliter range enable quantification of subtle preclinical changes, supporting application in early disease screening. More recently, fully automated platforms have demonstrated reproducibility across multi-center cohorts, reinforcing the feasibility of plasma Aβ as a scalable diagnostic biomarker [86,87]. However, variability related to pre-analytical handling, peripheral production sources and systemic inflammatory states remains an important consideration.

Given the increasing emphasis on early and minimally invasive diagnosis, a comparative evaluation of assay sensitivity and analytical performance is essential. Therefore, the performance characteristics of major Aβ detection platforms are summarized in Table 2. Unlike Table 1, which outlines general methodological principles, this table focuses on reported limits of detection (LOD/LOQ), biological matrices, and clinical applicability, providing a quantitative perspective on diagnostic reliability.

## 7. Conclusions and Future Directions

Despite significant advances, several critical knowledge gaps remain in our understanding of peripheral Aβ contributions to AD. First, the relative quantitative contribution of different peripheral sources such as platelets, liver, gut, and kidney to cerebral Aβ burden remains incompletely defined. Improved biomarker strategies are needed to distinguish central versus peripheral Aβ pools in vivo.

Second, the mechanisms governing intestinal Aβ production, transport, and interaction with the gut microbiota require further investigation. The gut–liver–brain axis represents an emerging frontier in AD research with potential diagnostic and therapeutic implications.

Third, while peripheral clearance pathways show promise as therapeutic targets, clinical validation is still limited. Large-scale longitudinal studies are needed to determine whether enhancing peripheral Aβ clearance can meaningfully alter disease trajectory in humans.

Finally, future therapeutic strategies may benefit from a combinatorial approach that integrates peripheral Aβ targeting with modulation of BBB function, neuroinflammation, and metabolic health. Viewing AD as a systemic disorder rather than a purely CNS-centric disease may open new avenues for early intervention and prevention.

## Figures and Tables

**Table 1:** Analytical tools and assay methods for investigating peripheral Amyloid β (Aβ). This table summarizes the various quantitative, structural, cellular, and functional techniques used to evaluate peripheral Aβ. It outlines the basis of each method alongside its respective advantages and limitations. Collectively, these tools offer complementary quantitative, structural, and mechanistic insights into peripheral Aβ dynamics that are relevant to biomarker development for AD.

Assay Method	Summary/Basis of Method	Advantages	Limitations	Manuscript References
Enzyme Immunoassay (ELS) and Electrochemiluminescence	Immunoassay-based quantitative detection of plasma Aβ_40_ and Aβ_42_.	Widely accessible; robust and reproducible detection of Aβ species.	Lower sensitivity compared to ultrasensitive platforms; limited detection at very low concentrations.	[35,36]
Single Molecule Array (Simoa)	Ultrasensitive digital immunoassay enabling detection of low-abundance plasma Aβ species.	Extremely high sensitivity (pg/mL range); suitable for early-stage detection.	Susceptible to pre-analytical variability; does not distinguish central vs. peripheral Aβ sources.	[36]
Immunoprecipitation Mass Spectrometry (IP-MS)	Combines antibody-based enrichment with mass spectrometry for precise quantification of Aβ_42_/Aβ_40_ ratio.	High analytical specificity and precision; gold standard for plasma Aβ ratio measurement.	Technically demanding; limited availability; similar biological limitations as Simoa.	[33]
Structural and Aggregation Analyses (Thioflavin-T binding, Circular Dichroism, Dynamic Light Scattering, Atomic Force Microscopy, Transmission Electron Microscopy)	Biophysical techniques used to assess Aβ conformation, aggregation kinetics, and fibril formation.	Enables characterization of oligomeric and β-sheet structures.	Requires purified samples; often non-physiological conditions; risk of preparation-induced artifacts.	[37,38,39,40]
Cellular Localization Assays (Immunohistochemistry, Confocal Imaging, Flow Cytometry)	Visualization and quantification of Aβ in platelets and PBMCs.	Provides spatial and cellular interaction data.	Dependent on antibody specificity; semi-quantitative; requires fresh samples (flow cytometry).	[41]
Functional Assays	Experimental systems assessing BBB transport and receptor-mediated Aβ clearance (e.g., LRP1, LDLR, RAGE).	Provides mechanistic insight into Aβ trafficking and clearance pathways.	Limited clinical validation; in vitro and animal models may not fully replicate human physiology.	[34]
Extracellular Vesicle Analysis	Isolation and analysis of EVs carrying neuron-derived Aβ in peripheral circulation.	Enables indirect assessment of CNS-derived Aβ; minimally invasive.	Lack of standardized protocols; technical challenges in EV isolation and purity.	[42]

**Table 2:** Analytical sensitivity and clinical applicability of major amyloid β detection methods, including reported limits of detection (LOD/LOQ) and representative references.

Method	Biological Sample	Target Output	Reported LOD/LOQ	Clinical/Research Relevance	Key References
ELISA/ECL	Plasma/CSF	Aβ_40_, Aβ_42_ concentration	~1–50 pg/mL	Established method for routine quantification; limited sensitivity for early-stage detection	[36]
Ultrasensitive immunoassays (e.g., SIMOA)	Plasma	Aβ_40_, Aβ_42_	~0.1–1 pg/mL	Enables detection in preclinical AD; suitable for large-scale screening	[36]
IP–MS	Plasma	Aβ_42_/Aβ_40_ ratio	pg/mL range (high precision)	High diagnostic accuracy; strong correlation with amyloid PET	[33]
CSF Assay	CSF	Aβ_42_ reduction, Aβ_42_/Aβ_40_ ratio	pg/mL range	Clinical gold standard	[36,43]
PET Imaging	Brain (in vivo)	Fibrillar amyloid deposition	Not applicable	Direct visualization of amyloid plaques	[43]
Extracellular Vesicles (EVs)	Plasma-derived EVs	Neuron-derived Aβ cargo	Not yet standardized	Emerging CNS-specific biomarker	[42]

## Data Availability

No new data were created or analyzed in this study. Data sharing is not applicable to this article.

## Abbreviations

AD	Alzheimer’s disease
Aβ	Amyloid β
Aβ_1–40_	Amyloid β 1–40
Aβ_1–42_	Amyloid β 1–42
APP	Amyloid precursor protein
BACE1	β-site APP-cleaving enzyme-1
BBB	Blood–brain barrier
RAGE	Receptor for advanced glycation end-products
LRP1	Low-density lipoprotein receptor-related protein-1
ABCA1	ATP-binding cassette transporter A1
ABCB1	ATP-binding cassette transporter B1 (P-glycoprotein)
CNS	Central nervous system
NAFLD	Non-alcoholic fatty liver disease
HDL	High-density lipoprotein
PET	Positron emission tomography
CSF	Cerebrospinal fluid
PBMC	Peripheral blood mononuclear cells
IP-MS	Immunoprecipitation mass spectrometry
SIMOA	Single molecule array
TEM	Transmission electron microscopy
ZO-1	Zonula occludens-1
ROS	Reactive oxygen species
TNF-α	Tumor necrosis factor-α
IL-1β	Interleukin-1β
IL-6	Interleukin-6
iNOS	Inducible nitric oxide synthase
DAMP	Danger-associated molecular pattern
ABAD	Amyloid-binding alcohol dehydrogenase
NEP	Neprilysin
ACE	Angiotensin-converting enzyme
PPARγ	Peroxisome proliferator-activated receptor-γ
SREBP-2	Sterol regulatory element-binding protein-2
EVs	Extracellular vesicles
ARIA	Amyloid-related imaging abnormalities
